# Long-Term Resistance of *Drosophila melanogaster* to the Mushroom Toxin Alpha-Amanitin

**DOI:** 10.1371/journal.pone.0127569

**Published:** 2015-05-15

**Authors:** Chelsea L. Mitchell, Roger D. Yeager, Zachary J. Johnson, Stephanie E. D’Annunzio, Kara R. Vogel, Thomas Werner

**Affiliations:** 1 Department of Biological Sciences, Michigan Technological University, Houghton, Michigan, United States of America; 2 Department of Experimental and Systems Pharmacology, Washington State University, Spokane, Washington, United States of America; Alexander Fleming Biomedical Sciences Research Center, GREECE

## Abstract

Insect resistance to toxins exerts not only a great impact on our economy, but also on the ecology of many species. Resistance to one toxin is often associated with cross-resistance to other, sometimes unrelated, chemicals. In this study, we investigated mushroom toxin resistance in the fruit fly *Drosophila melanogaster* (Meigen). This fruit fly species does not feed on mushrooms in nature and may thus have evolved cross-resistance to α-amanitin, the principal toxin of deadly poisonous mushrooms, due to previous pesticide exposure. The three Asian *D*. *melanogaster* stocks used in this study, Ama-KTT, Ama-MI, and Ama-KLM, acquired α-amanitin resistance at least five decades ago in their natural habitats in Taiwan, India, and Malaysia, respectively. Here we show that all three stocks have not lost the resistance phenotype despite the absence of selective pressure over the past half century. In response to α-amanitin in the larval food, several signs of developmental retardation become apparent in a concentration-dependent manner: higher pre-adult mortality, prolonged larva-to-adult developmental time, decreased adult body size, and reduced adult longevity. In contrast, female fecundity nearly doubles in response to higher α-amanitin concentrations. Our results suggest that α-amanitin resistance has no fitness cost, which could explain why the resistance has persisted in all three stocks over the past five decades. If pesticides caused α-amanitin resistance in *D*. *melanogaster*, their use may go far beyond their intended effects and have long-lasting effects on ecosystems.

## Introduction

Insect pesticide resistance costs the United States billions of dollars in crop losses and pesticide design every year [1]. Oftentimes, pesticide resistance is associated with cross-resistance to several other chemicals, such as in mosquitoes [2,3], potato beetles [4], whiteflies [5], diamondback moths [6], cockroaches [7], house flies [8], and fruit flies [9–11]. In this study, we describe a curious mushroom toxin resistance phenotype in the fruit fly *D*. *melanogaster*, which may have evolved from pesticide exposure in their natural habitats.

α-Amanitin is the principal toxin of several deadly poisonous mushrooms, such as the Death Cap and Destroying Angel [12]. These two mushroom species account for about 90% of the mushroom-related deaths in the United States [13]. α-Amanitin exerts its toxic function by inhibiting RNA-polymerase II, thereby interfering with messenger RNA production in eukaryotic organisms [14]. Because RNA-polymerase II in all tested wild-caught fruit fly species is very susceptible to this toxin [15,16], the flies must employ unique mechanisms that prevent the toxin from entering the nucleus of the cells, where the RNA-polymerase II is active.

Mushroom-feeding (mycophagous) *Drosophila* species are super-resistant to all mushroom toxins, allowing them to breed in virtually all toxic mushrooms [15–18]. This unusual ability provides these flies with access to a unique food source and protection from parasitic nematodes, which would render the flies sterile [19,20].

Paradoxically, mushroom toxin resistance is even found in some mushroom-avoiding fruit flies, such as certain stocks of the genetic model organism *D*. *melanogaster* [21,22]. Because α-amanitin is solely produced by mushrooms [23–25], these flies should never encounter this mushroom toxin in nature. In the 1960s, the first three α-amanitin-resistant *D*. *melanogaster* stocks were isolated in Asia: Ama-KTT from Taiwan, Ama-MI from India, and Ama-KLM from Malaysia. In 1982, they were shown to be 29-fold, 25-fold, and 8.3-fold, respectively, more resistant to α-amanitin than the susceptible wild type stock Oregon-R [22]. These three resistant stocks are, however, not resistant enough to survive a poisonous mushroom diet [22,26].

In two studies, α-amanitin resistance of four resistant Asian and North American *D*. *melanogaster* stocks was mapped to virtually the same two dominantly acting loci on the third chromosome [21,22], suggesting that the resistance phenotype may have spread globally. Begun and Whitley [21] suggested that the Multidrug resistance pump gene *Mdr65* (on the left arm of chromosome 3) and the *Protein kinase C98E* (*Pkc98E*) gene (on the right arm of chromosome 3) confer α-amanitin resistance, thus, protecting the susceptible RNA-polymerase II in the nucleus. In our previously published work, we re-investigated this case by performing a microarray analysis, using the Ama-KTT-derived isochromosomal line Ama-KTT/M/2. We found that four molecular mechanisms, but probably not a multidrug resistance pump, may contribute to α-amanitin resistance in this *D*. *melanogaster* stock: cuticular proteins block the entry of α-amanitin into cells, Cytochrome P450 and Glutathione-S-transferase enzymes detoxify α-amanitin, peptidases cleave α-amanitin, and lipid particles sequester α-amanitin in the cytoplasm [26]. Remarkably, three Cytochrome P450 genes were at least 200-fold constitutively up-regulated in the resistant larvae: *Cyp6a2*, *Cyp12d1-d*, and *Cyp12d1-p*. These genes have been shown to respond to, or detoxify, various chemically unrelated substances, including the pesticides DDT, imidacloprid, dicyclanil, atrazine, and the drug phenobarbital [27–32]. Thus, α-amanitin resistance in *D*. *melanogaster* may have evolved as cross-resistance to pesticides applied to the habitats of these flies, such as gardens, vineyards, and other fruit plantations.

In the present study, we show that the three Asian *D*. *melanogaster* stocks Ama-KTT, Ama-MI, and Ama-KLM are still resistant to α-amanitin, even after five decades of being maintained in a stock center without any selective pressure (~1,200 generations). Furthermore, the addition of α-amanitin to the larval food increases female fecundity, but also affects larva-to-adult development and longevity of the resistant fly stocks. We conclude that α-amanitin resistance has no obvious fitness costs in the three Asian *D*. *melanogaster* stocks, explaining why the resistance phenotype has persisted in these populations for such a long time.

## Results

### After five decades without selective pressure, the three Asian fly stocks are still resistant to α-amanitin

**Fig 1:**
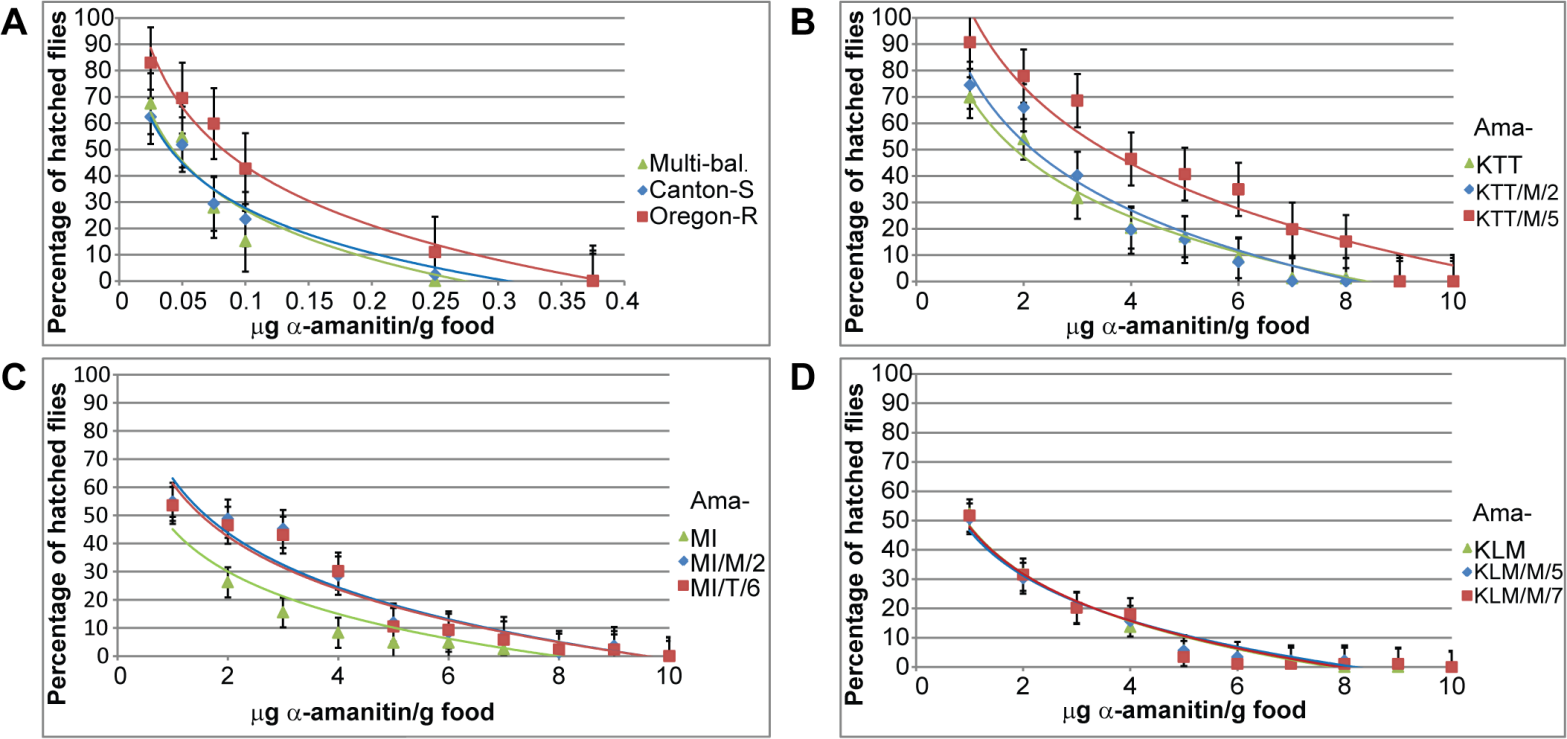
LC_50_ analyses for all fly stocks. A) Oregon-R, Canton-S, and multi-balancer stock; B) Ama-KTT, Ama-KTT/M/2, and Ama-KTT/M/5; C) Ama-MI, Ama-MI/M/2, and Ama-MI/T/6; D) Ama-KLM, Ama-KLM/M/5, and Ama-KLM/M/7 LC_**50**_ analyses are shown. All analyses contain three experimental replicates (100 larvae in each experiment for each concentration) and were normalized, using 0-toxin concentration as a control. The error bars represent the standard error of the mean (s.e.m.).

The three Asian *D*. *melanogaster* stocks Ama-KTT from Taiwan, Ama-MI from India, and Ama-KLM from Malaysia were collected from their natural habitats in the 1960s. In 1982, i.e. two decades after their isolation, these stocks were shown to be 29-fold (Ama-KTT), 25-fold (Ama-MI), and 8.3-fold (Ama-KLM) more resistant to the mushroom toxin α-amanitin than the susceptible wild type stock Oregon-R [22]. In this study, we tested if these three Asian fly stocks have retained their resistance after five decades of being reared in the stock center without selective pressure. We first calculated the current lethal concentration 50 (LC_50_) values of Ama-KTT, Ama-MI, Ama-KLM, and Oregon-R, which are the α-amanitin concentrations in the larval food in [μg/g] that cause 50% of the individuals to die before the adults emerge. Additionally, we included the wild type stock Canton-S in our comparison because it has recently become a more widely used control in many studies. For each dose-response curve, we placed 100 freshly hatched first-instar larvae per concentration on α-amanitin-containing food. Eleven toxin concentrations (including the 0-toxin control) were used, and three replicates were performed for each dose-response experiment. We counted hatching flies as survivors, followed by ANOVA analysis. From this experiment, we established that all three Asian fly stocks are still more resistant than Oregon-R: Ama-KTT is currently 22-fold, Ama-MI 10-fold, and Ama-KLM 11-fold more resistant than the Oregon-R control flies (Fig 1, Table 1). We note that the resistance differences observed between 1982 and today may be due to the slightly different methodologies used in both studies: for higher accuracy, we manually placed healthy, counted first-instar larvae on toxic food, while in the 1982 study, females laid uncontrolled numbers of eggs on non-toxic food that was later supplemented with α-amanitin.

**Table 1:** Comparison between current and historic resistance values.

Stock	LC_50_ (± s.e.m)	Current comparison to Oregon-R	1982 Comparison to Oregon-R
Canton-S	0.028 (± 0.001)	0.34-fold	-
Oregon-R	0.082 (± 0.005)	-	-
Multi-balancer	0.042 (± 0.001)	0.51-fold	-
Ama-KTT	1.843 (± 0.054)	22-fold	29-fold
Ama-KTT/M/2	2.167 (± 0.074)	26-fold	-
Ama-KTT/M/5	3.522 (± 0.120)	43-fold	-
Ama-MI	0.797 (± 0.094)	10-fold	25-fold
Ama-MI/M/2	1.600 (± 0.038)	20-fold	-
Ama-MI/T/6	1.518 (± 0.035)	19-fold	-
Ama-KLM	0.924 (± 0.052)	11-fold	8.3-fold
Ama-KLM/M/5	0.855 (± 0.052)	10-fold	-
Ama-KLM/M/7	0.912 (± 0.057)	11-fold	-

Our calculated LC_50_ values and how they compare to the values calculated in 1982 [22] are shown. Oregon-R served as the normalization control for the relative resistance values between today and 1982. LC_50_ values are given in [μg of α-amanitin per g of larval food]. All values are averages of three experimental replicates.

We further investigated the α-amanitin resistance level of the commonly used control stock Canton-S. Our data show that Canton-S is three times more susceptible to the toxin than Oregon-R (Fig 1A, Table 1). Comparing the various resistance levels of all five stocks that we tested, it seems that α-amanitin resistance is a more variable genetic trait among *D*. *melanogaster* stocks than it was previously anticipated.

**Fig 2:**
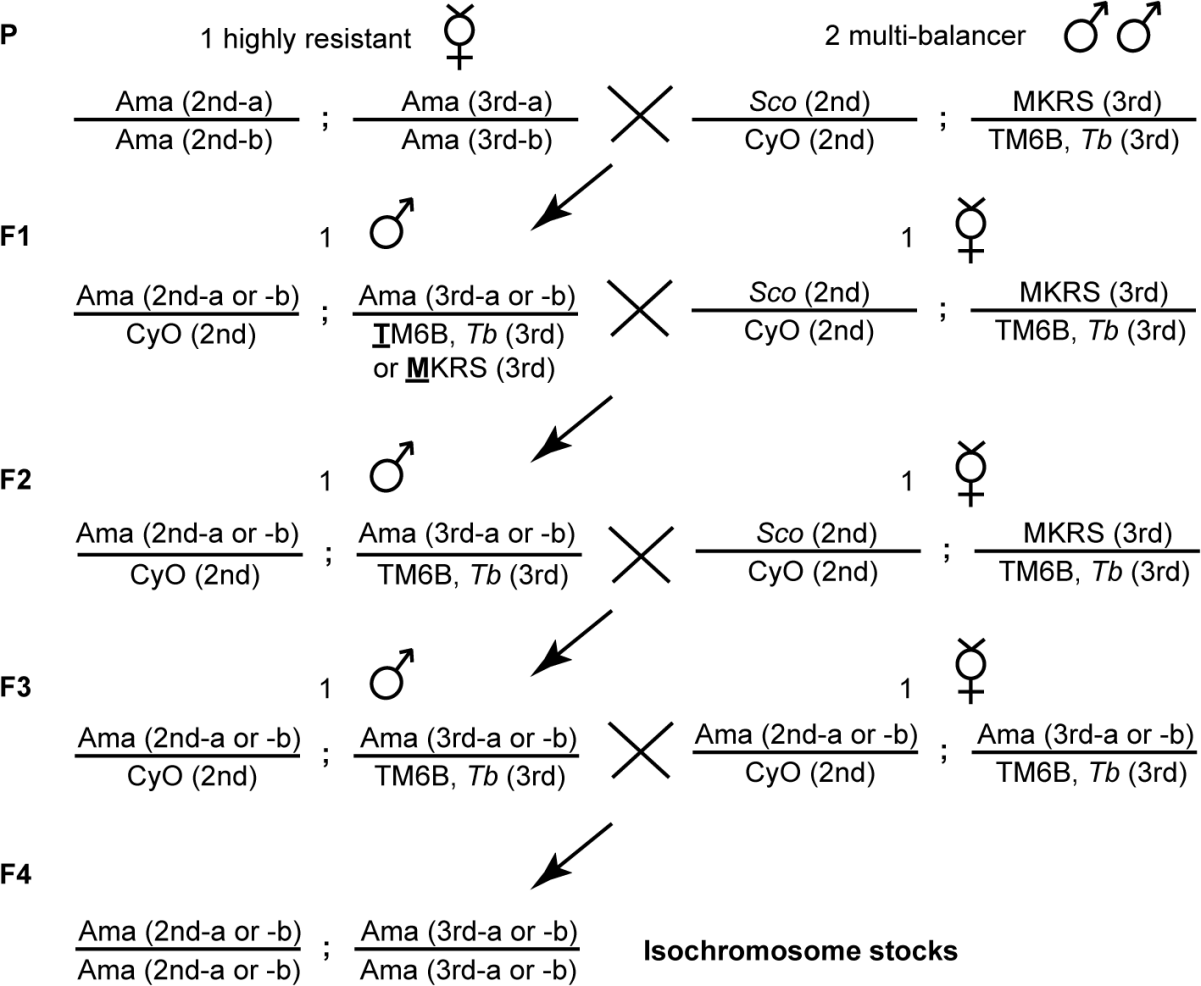
Crossing scheme for the generation of the isochromosomal lines. One highly resistant virgin female of each original Asian fly stock was mated with two males of the multi-balancer stock. F1 generation males that carried an Ama chromosome 2 balanced over CyO and an Ama chromosome 3 balanced over TM6B, *Tb* or MKRS were crossed back to one multi-balancer virgin female. F2 generation males carrying an Ama chromosome 2 balanced over CyO and an Ama chromosome 3 balanced over TM6B, *Tb* were back-crossed to one multi-balancer virgin female. Virgin siblings of the F3 generation were then crossed to produce the isochromosomal lines.

Over the past ~50 years, allelic drift and/or reverse mutations of resistance-conferring alleles could have occurred in the stock center. Therefore, we wanted to make sure that the three Asian stocks are still largely homozygous for the resistance-conferring alleles/loci. We thus created isochromosomal lines by using one toxin-selected, highly resistant virgin female of Ama-KTT, Ama-MI, and Ama-KLM, following the crossing scheme outlined in Fig 2. Although Phillips et al. [22] suggested that only two dominantly acting third chromosome loci underlie α-amanitin resistance in all three Asian fly stocks, we did not exclude the possibility that genes located on other chromosomes contribute to the resistance. Thus, we created isochromosomal lines that are isogenic for both major autosomes: chromosomes 2 and 3. We preliminarily tested all resulting isochromosomal lines for α-amanitin resistance, with the result that all of them were approximately as resistant to the toxin as the original stocks (Fig 1, Table 1). We then focused on two randomly chosen isochromosomal lines that descended from each original Asian stock (Ama-KTT/M/2, Ama-KTT/M/5, Ama-MI/M/2, Ama-MI/T/6, Ama-KLM/M/5, and Ama-KLM/M/7) and calculated their exact LC_50_ values. As a result, the isochromosomal lines showed similar resistance levels to their parental stocks, suggesting that no major genetic changes have reversed the resistance phenotype over time. We note that the small differences that we detected in our assay may be due to experimental noise.

### α-Amanitin delays larva-to-adult development in a concentration-dependent manner

**Fig 3:**
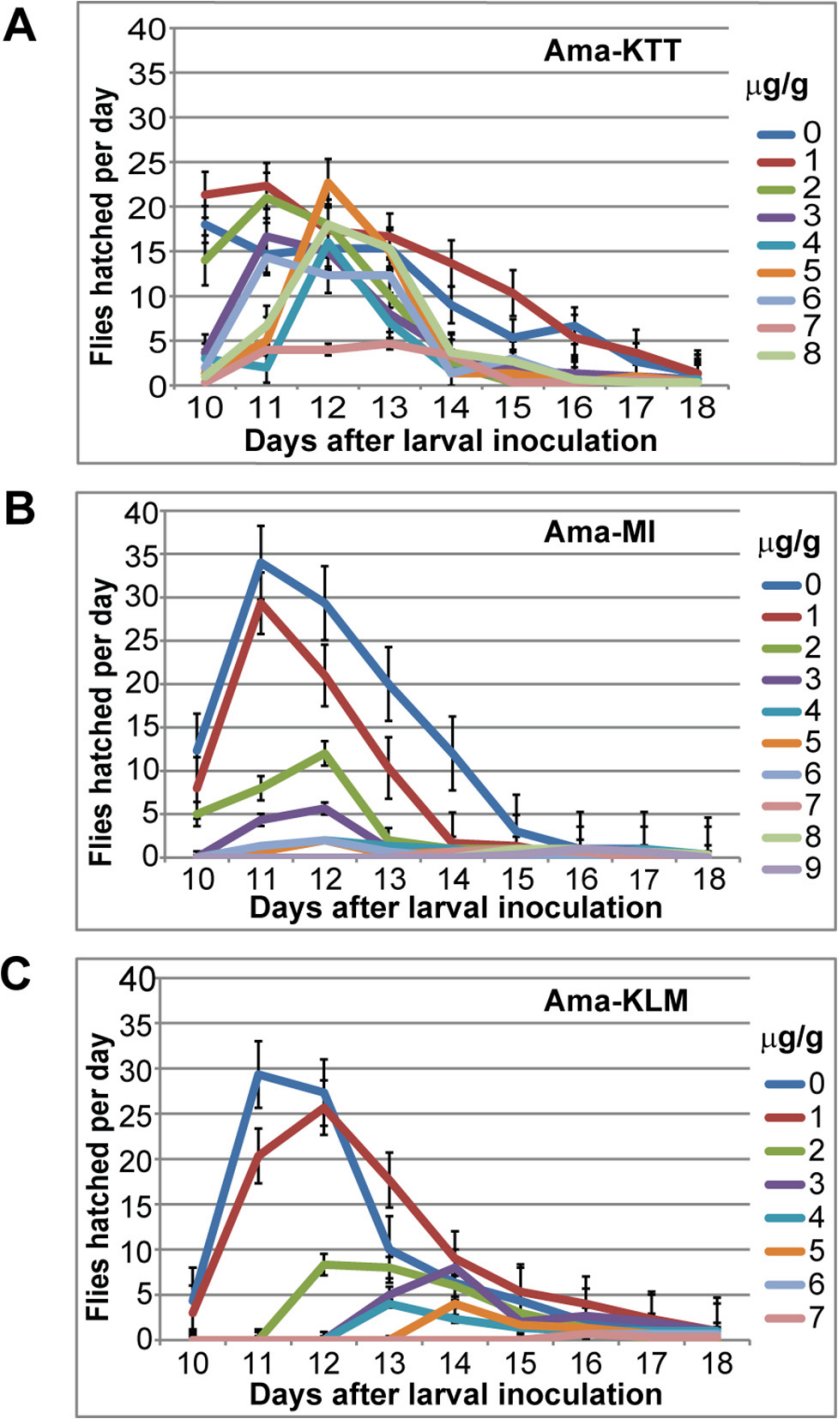
Adult hatch time delay of the three original Asian stocks. A) Ama-KTT, B) Ama-MI, and C) Ama-KLM. The first-instar larvae were laid on day 0. The data resulting from three experimental replicates were pooled. The error bars represent the s.e.m.

Mycophagous *Drosophila* species are usually super-resistant to mushroom toxins and show no deleterious developmental effects when breeding in most toxic mushrooms. Only at extremely high α-amanitin concentrations (250–1000 μg α-amanitin per g of mushroom), some mycophagous *Drosophila* species can show signs of developmental retardation, i.e., the larvae develop more slowly and the adults are smaller and have sometimes reduced or missing eyes [17]. We were curious to see if the three resistant Asian *D*. *melanogaster* stocks Ama-KTT, Ama-MI, and Ama-KLM show similar developmental retardation symptoms in response to increasing α-amanitin concentrations and at what toxin concentrations these symptoms become apparent. First, we investigated the effect of α-amanitin on the larva-to-adult developmental time of Ama-KTT, Ama-MI, and Ama-KLM. For these experiments, we used the same animals that gave rise to the LC_50_ data, followed by ANOVA analysis. Once every day, we recorded the numbers of hatched flies from each toxin concentration. We then compared the days on which the hatching activity peaked. Our results (Fig 3) show that all three Asian stocks behaved similarly: increased α-amanitin concentrations caused concentration-dependent hatch time delays. For all three fly stocks, the lowest toxin concentrations delayed the peak of fly hatching by one day, while the highest tolerable concentrations caused up to three days of hatch delay, as compared to the 0-toxin concentration. Thus, unlike mycophagous *Drosophila* species, the three resistant Asian *D*. *melanogaster* stocks showed a developmental retardation phenotype that became apparent even at low toxin concentrations and became more severe as the toxin concentrations increased.

### α-Amanitin reduces adult body size development in a concentration-dependent manner

**Fig 4:**
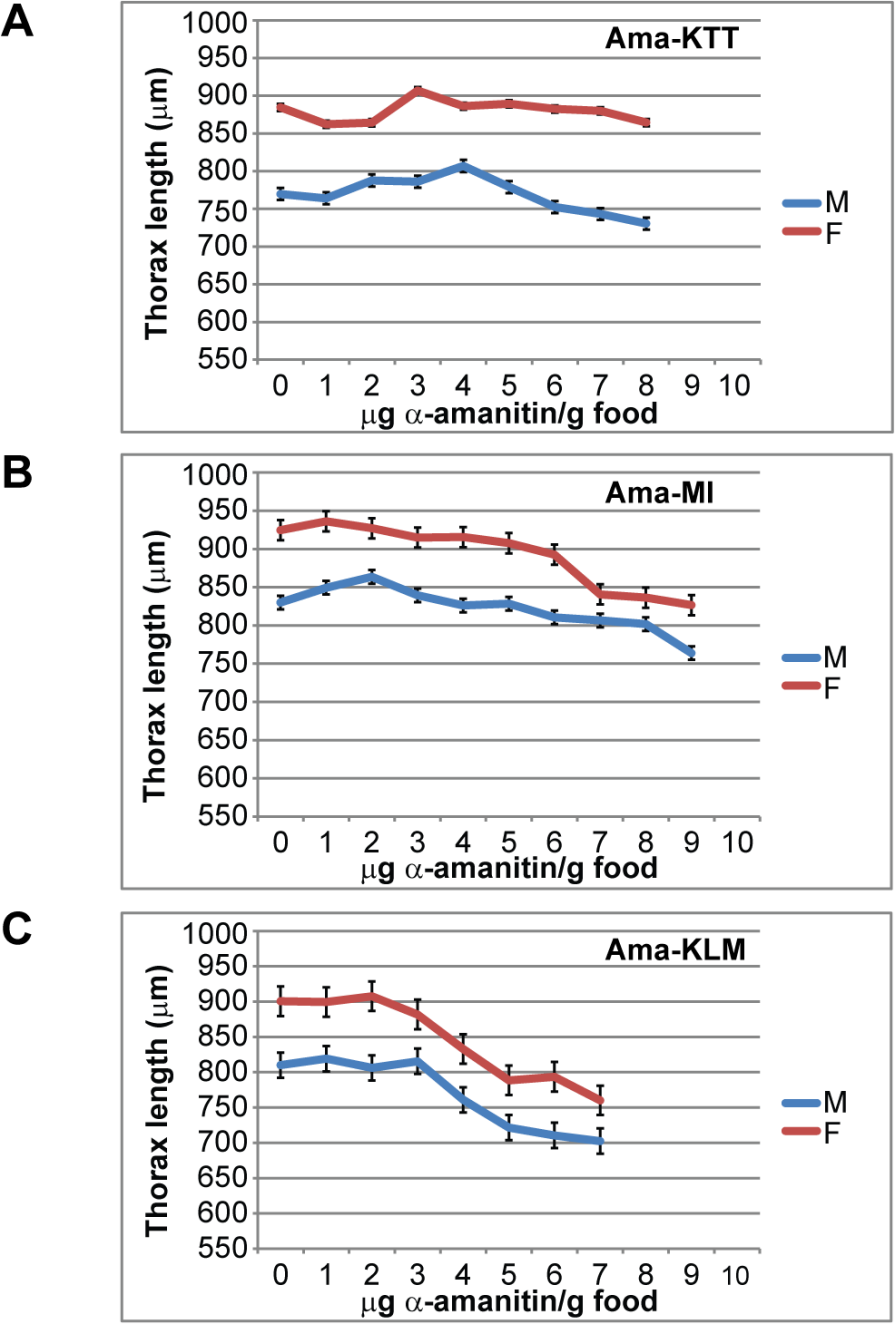
Adult thorax lengths of the three original Asian stocks. A) Ama-KTT, B) Ama-MI, and C) Ama-KLM. Male and female thorax lengths (y-axis) from flies that hatched from different α-amanitin concentrations (x-axis) were measured. The data resulting from three experimental replicates were pooled. The error bars represent the s.e.m.

We then tested how α-amanitin affects the adult body size of the three Asian stocks Ama-KTT, Ama-MI, and Ama-KLM (Fig 4), using again the same flies that were used in the previous LC_50_ and hatching time analyses. Because thorax lengths of flies are fixed throughout life and directly correlate with overall body size [33–37], we measured the thorax lengths of all of the flies as a proxy for overall body size. Each experiment was performed in three replicates, and the data underwent ANOVA analysis. Our results show that all three Asian *D*. *melanogaster* stocks responded in similar ways to increasing α-amanitin concentrations, but differently from how mycophagous species respond to the same toxin. We observed seven trends that the three resistant *D*. *melanogaster* stocks shared (Fig 4): 1) on toxin-free control food, the emerging flies were somewhat smaller than flies that hatched from the lowest α-amanitin concentrations. 2) With increasing toxin concentrations, the thorax lengths first increased until a "sweet spot" was reached, which was always slightly above the LC_50_ of the respective stock (Fig 4, Table 1). This paradoxical thorax length increase may be an indirect effect due to reduced larval crowding, so that the surviving larvae had more food and could grow larger. 3) Above the "sweet spot" concentration, the thorax lengths then started to gradually decline in a toxin concentration-dependent manner. 4) In all three Asian *D*. *melanogaster* stocks, the female's onset of thorax length decline started exactly at one concentration increment lower than in males, indicating that males may be slightly more resistant to α-amanitin than females. 5) The highest tolerable toxin concentration of each stock always resulted in thorax lengths lower than those at the 0-toxin concentration. 6) The higher the LC_50_ of a stock, the more α-amanitin was necessary to bring the thorax length values below that of the 0-toxin concentration. 7) The lower the LC_50_ of a stock, the further the thorax lengths declined below the values of the 0-toxin concentration. In summary, *D*. *melanogaster's* body size is affected by α-amanitin in a gradual, concentration-dependent manner, which stands in contrast to the sudden response in mycophagous flies at only the highest tolerable toxin concentrations. Furthermore, none of the three Asian *D*. *melanogaster* stocks showed signs of reduced or missing eyes on any α-amanitin concentration. The resistance of the three Asian *D*. *melanogaster* stocks is, although impressive compared to other susceptible stocks of this species, still two to three orders of magnitude weaker than the resistance of mycophagous flies.

### α-Amanitin in the larval food increases egg-lay performance in adult females

**Fig 5:**
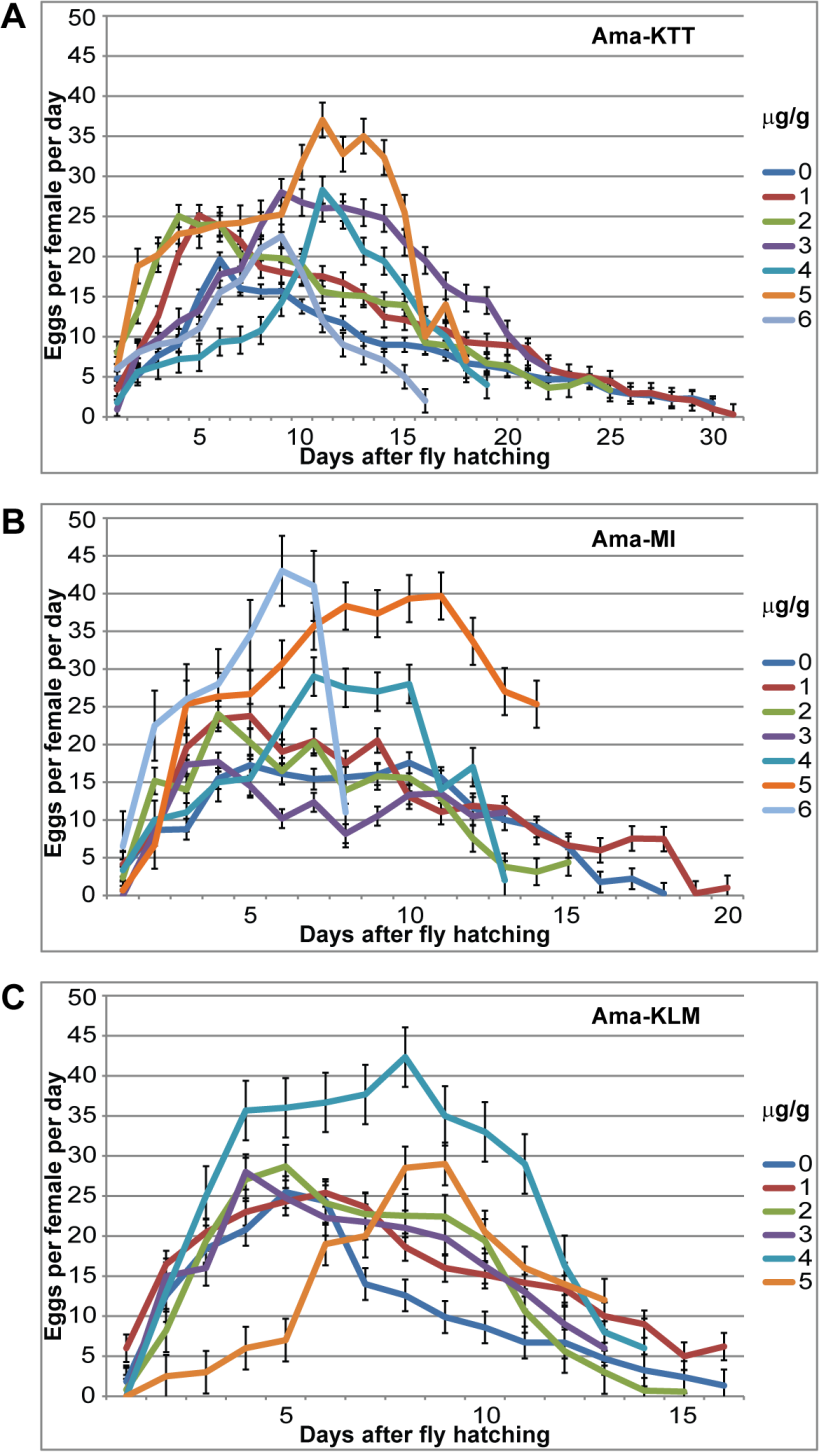
Female fecundity of the three original Asian stocks. A) Ama-KTT, B) Ama-MI, and C) Ama-KLM. Day 0 is the day of adult female hatching. The data resulting from three experimental replicates were pooled. The error bars represent the s.e.m.

For the remaining tests, adults from the previous analyses that hatched within the three days of peak hatching were kept alive on non-toxic molasses agar and fresh yeast in egg-lay chambers until they died. The flies were supplied with fresh food on a daily basis. We next asked the questions if and how different α-amanitin concentrations fed to the larvae affect female fecundity of the hatched flies. We grouped all females that hatched on the same day (day 0) from each toxin concentration into one egg-lay chamber and monitored their fecundity daily. Because females have a shorter lifespan when males are present [38], all females were accompanied by an equal number of males to balance the sex ratio across all experiments. When available, we added males of the same stock that hatched on the same day from the same toxin concentration. As an alternative, we accompanied our experimental females with young white-eyed males of the *w* ^1118^ stock because they could be easily distinguished from the toxin-resistant flies and thus excluded from the longevity experiments, as described in the next section. We performed three experimental replicates, and the data underwent ANOVA analysis. Considering the negative effects that α-amanitin exerts on the development of the three Asian *D*. *melanogaster* stocks, we expected that higher toxin concentrations would result in lower eggs-per-female production rates and delayed egg-lay peak times. All three Asian stocks responded in a similar manner to increasing α-amanitin concentrations (Fig 5, Table 2). In contrast to our expectation, at the two to three lowest toxin concentrations, the egg-lay peak performance was shifted to one day *earlier* than that of the 0-toxin concentration flies. Often, the flies on these toxin concentrations also laid *more* eggs than on the 0-toxin concentration. The higher α-amanitin concentrations then caused the expected concentration-dependent delay in egg-lay activity peaks by up to four days. Perhaps the most surprising result was that each stock produced about twice the amount of eggs per female at the second highest tolerable α-amanitin concentration, as compared to the 0-toxin concentration (Table 2). Our results indicate that α-amanitin increases the reproductive fitness of all three Asian fly stocks.

**Table 2:** Average total egg productivity of the three original Asian stocks in response to α-amanitin in the larval food.

Stock	α-Amanitin concentration	Average total eggs/female (± s.e.m.)
Ama-KTT	0	72.17 (± 0.89)
	1	96.28 (± 1.29)
	2	93.46 (± 1.41)
	3	87.95 (± 1.67)
	4	50.37 (± 1.69)
	5	109.73 (± 2.17)
	6	53.00 (± 1.48)
Ama-MI	0	58.88 (± 1.38)
	1	75.60 (± 1.63)
	2	54.19 (± 1.77)
	3	37.94 (± 1.26)
	4	49.67 (± 2.55)
	5	130.89 (± 3.13)
	6	70.83 (± 4.64)
Ama-KLM	0	33.17 (± 1.99)
	1	79.28 (± 1.71)
	2	59.38 (± 2.70)
	3	55.93 (± 2.19)
	4	86.75 (± 3.71)
	5	57.17 (± 2.66)

The average total eggs-per-female numbers for the three original Asian stocks in response to different α-amanitin concentrations in [μg of α-amanitin per g of larval food] are shown. All values are averages of three experimental replicates.

### α-Amanitin in larval food exerts a negative long-term effect on adult lifespan

We further tested if α-amanitin in the larval food affects the longevity of our flies in the egg-lay chambers. We performed three replicates for each experiment, and the data underwent ANOVA analysis. As expected, all three Asian stocks responded with reduced adult lifespans in response to larval food containing increasing amounts of α-amanitin (Table 3). We also noted that males of all three stocks survived longer than females in both the presence and absence of toxin. Interestingly, at the two to three highest tolerable α-amanitin concentrations for each stock, the females died almost immediately after they hatched, while males at these concentrations lived for about a week. This observation was the second indication that males of all three stocks may be more resistant than females. Furthermore, our most resistant stock Ama-KTT also showed the highest overall lifespan, while the two less resistant stocks Ama-MI and Ama-KLM had about 30% shorter lifespans. This observation held true for both sexes with and without the toxin. For example, Ama-KTT males that were raised as larvae on non-toxic food lived 32.33 (±2.03) days, while the less resistant Ama-MI and Ama-KLM males only lived for 24.33 (±1.20) and 22.33 (±1.45) days, respectively (Table 3). We note that although the most resistant Asian stock has the longest life expectancy, many factors can determine lifespan, such as different genetic backgrounds [39,40]. Therefore, we cannot conclude that high resistance correlates with high life expectancy.

**Table 3:** Longevity of the three original Asian stocks.

α-Amanitin concentration	Sex	Ama-KTT lifespan (± s.e.m.)	Ama-MI lifespan (± s.e.m.)	Ama-KLM lifespan (± s.e.m.)
0	M	32.33 (± 2.03)	24.33 (± 1.20)	22.33 (± 1.45)
	F	29.00 (± 1.53)	17.33 (± 1.45)	15.67 (± 0.33)
1	M	27.33 (± 0.33)	24.67 (± 1.20)	21.33 (± 1.20)
	F	25.00 (± 1.15)	15.67 (± 1.45)	14.67 (± 0.88)
2	M	24.67 (± 0.88)	19.33 (± 0.88)	16.00 (± 0.58)
	F	24.00 (± 1.15)	13.67 (± 0.88)	14.00 (± 0.58)
3	M	18.67 (± 0.88)	16.67 (± 0.88)	14.67 (± 0.88)
	F	15.33 (± 1.20)	12.33 (± 0.67)	12.67 (± 0.33)
4	M	19.33 (± 1.45)	12.67 (± 0.88)	13.33 (± 0.88)
	F	18.33 (± 2.03)	13.33 (± 0.88)	12.00 (± 1.15)
5	M	15.67 (± 1.20)	11.67 (± 1.45)	11.33 (± 0.67)
	F	12.67 (± 0.88)	9.00 (± 1.15)	9.67 (± 1.76)
6	M	13.33 (± 1.86)	10.67 (± 0.88)	11.00 (± 0.58)
	F	8.67 (± 1.45)	6.33 (± 0.88)	Instant death
7	M	11.33 (± 0.88)	8.67 (± 0.67)	6.00 (± 0.58)
	F	Instant death	Instant death	Instant death
8	M	8.67 (± 0.88)	6.67 (± 0.33)	-
	F	Instant death	Instant death	-
9	M	-	5.67 (± 0.33)	-
	F	-	Instant death	-
10	M	-	-	-
	F	-	-	-

The average lifespans of males (M) and females (F) for for the three original Asian stocks in response to different α-amanitin concentrations in [μg of α-amanitin per g of larval food] are shown. All adult lifespan values are given in days and are averages of three experimental replicates.

## Discussion

### α-Amanitin resistance has no apparent fitness cost

One of the most intriguing aspects of *D*. *melanogaster*'s α-amanitin resistance is that the toxin is exclusively found in mushrooms [12], whereas the flies are not attracted to mushrooms and should not encounter α-amanitin in nature. Therefore, the resistance appears to be a cross-resistance to other toxic compounds that the flies encountered in their Asian habitats at least 50 years ago. We show that five decades after their isolation, the three Asian *D*. *melanogaster* stocks Ama-KTT, Ama-MI, and Ama-KLM are still more resistant to α-amanitin than the susceptible wild type stocks Oregon-R and Canton-S. Comparing the combined LC_50_ data of all analyzed stocks in this study, our data strongly suggest that α-amanitin resistance is conferred by many genes with smaller effects, as opposed to only two dominant alleles on the third chromosome alone, as it was suggested by Phillips et al. [22]. This conclusion is further supported by our previous microarray study [26], in which we showed that several candidate genes and molecular mechanisms may be collectively contributing to the α-amanitin resistance phenotype of the isochromosomal line Ama-KTT/M/2. Notably, three *Cyp* genes were among the resistance-conferring candidate genes of Ama-KTT/M/2, which have been associated with pesticide resistance and stress responses. It is therefore very likely that α-amanitin resistance in the three Asian *D*. *melanogaster* stocks is a cross-resistance to agricultural pesticides that the flies encountered in the wild at least 50 years ago. Due to the fact that the resistance phenotype has persisted over such a long time, α-amanitin resistance seems to have no major fitness costs. In a similar example, *Cyp6g1*-mediated DDT resistance in *D*. *melanogaster* also has no fitness cost, which caused the resistance-conferring *DDT-R* allele to reach global fixation even after the use of DDT was banned [41,42].

### α-Amanitin causes developmental retardation phenotypes that resemble stress responses

When we fed increasing concentrations of α-amanitin to resistant larvae, we observed the following four detrimental developmental effects in all the three Asian stocks: 1) higher pre-adult mortality, 2) prolonged larva-to-adult developmental time, 3) decreased adult body size, and 4) reduced adult longevity. The severity of the retardation symptoms was inversely correlated with the LC_50_ values to the toxin; i.e., the more resistant a stock, the less affected it was by α-amanitin.

Our data show that pre-adult mortality and larva-to-adult developmental time increased in an α-amanitin concentration-dependent manner. In a similar study, *D*. *melanogaster* larvae that were fed with the mushroom toxin ibotenic acid also showed reduced pre-adult survivorship and prolonged developmental time [43]. The observed developmental retardation of larvae that feed on toxic food suggests that the detoxification processes take essential resources away from development, thereby slowing growth.

Adult body size was also affected by α-amanitin in a concentration-dependent manner. However, we noted a paradoxical increase in adult thorax lengths at the lowest toxin concentrations in all three Asian stocks, which may be explained by the fact that adult body size is affected by larval crowding in many insect species [44]. Thus, the unexpected increase in body size on low α-amanitin concentrations could be attributed to the reduced larval crowding conditions as some larvae die from the toxin. Several other studies show that thorax lengths also decrease in response to other toxins, stress, and parasitism in *D*. *melanogaster*, e.g. the mushroom toxin ibotenic acid [43], temperature stress [34], and hymenopteran parasitoid attack [36].

When the larvae were reared on α-amanitin-containing food, adult longevity showed a negative correlation to increasing α-amanitin concentrations in the larval food, i.e., the longevity decreased in all stocks in a toxin concentration-dependent manner. These results suggest that some α-amanitin might remain in the hatched flies and affect adult longevity. This observation may be due to one of our previously suggested detoxification mechanisms, which is that the larvae sequester parts of the ingested α-amanitin in the body [26].

In all three Asian fly stocks, adult longevity was higher in males than in females under all conditions. We further observed that at the two to three highest toxin concentrations, all females died almost instantly after they hatched, while the males lived for about one week. This observation could either mean that males are more toxin-resistant than females, or that females generally have shorter lifespans. Norry et al. [45] showed that heat-stressed males of *D*. *melanogaster* live longer than heat-stressed females. Furthermore, different stress factors have been shown to reduce longevity in both sexes of *Drosophila*, e.g. stress caused by microsporidian infection [46] and higher temperature [37,47].

### α-Amanitin increases female fecundity

Exposure to low α-amanitin concentrations caused an earlier onset of female fecundity and an increase in the eggs-per-female rate in all three Asian stocks. The most dramatic increase in fecundity was observed at the second highest tolerable toxin concentration for each stock. Although the peak egg-lay time was delayed by several days at this concentration (Fig 5), the average total eggs-per-female productivity roughly doubled (Table 2). A possible explanation for the dramatic fecundity increase is that α-amanitin is sequestered in the hatched adults, causing stress responses that alter female fecundity and egg-lay behavior. Similar examples are known, where life expectancy-lowering stress factors increase female fecundity. For example, physical injury can cause female moths to lay their eggs faster and on less suitable substrates than non-injured control moths [48]. Furthermore, stress caused by pathogens and parasitoids can also increase female fecundity in insects, e.g. in crickets [49].

Another surprising outcome of our study is that α-amanitin resistance seems to have no obvious fitness costs, which is in contrast to several other studies addressing insect resistance to various factors, such as the resistance of *Drosophila* to microsporidian pathogens [46] and hymenopteran parasitoides [36], that of brown planthoppers to a pesticide [50], of mosquitoes to malaria parasites [51], and the resistance of snails to schistosome parasites [52]. Fitness costs can be determined by the resistance-conferring genes and/or the environment. For example, in mosquitoes, the cost of resistance to organophosphates can range from no cost to very high costs, depending on the resistance-conferring genes [53,54]. In moths, resistance to *Bacillus thuringiensis* toxins has fitness costs especially when the animals are stressed or parasitized [55,56]. However, when conferred by the *DDT-R* locus, the resistance of *D*. *melanogaster* to the pesticide DDT has no apparent fitness costs but instead benefits [41,42]. Interestingly, similar to the Asian α-amanitin-resistant fly stocks, the *DDT-R* allele-carrying flies show an increased viability and female fecundity.

### Implications

The implications of our study, which is the most detailed phenotypic analysis of naturally occurring α-amanitin-resistant *D*. *melanogaster* stocks to date, are two-fold: 1) *D*. *melanogaster* displays several stress-like responses to the complete range of sub-lethal α-amanitin concentrations, while mycophagous species remain unaffected by most sub-lethal concentrations [15,17]. The observed physiological differences between mycophagous and non-mycophagous *Drosophila* species suggest that different molecular-genetic mechanisms underlie α-amanitin-resistance in ecologically distinct species. 2) The increased fecundity of resistant *D*. *melanogaster* females in response to α-amanitin may have important implications on the interactions of this species with its environment: if resistant females would change their egg-lay preferences to include, for example, mushrooms, *D*. *melanogaster* may be well-prepared for invading the toxic mushroom niche and begin to feed on specimens with low toxicity, perhaps evolving higher resistance over time. *D*. *melanogaster* is already capable of completing its life cycle solely on non-toxic fungi, e.g. Baker's yeast, in the laboratory. This scenario of a non-mycophagous species entering the toxic mushroom niche is not entirely hypothetical, as ecologically intermediate species do exist. For example, *Drosophila tripunctata* feeds on both fermenting fruit and mushrooms [57,58]. While *D*. *tripunctata* is much more resistant to α-amanitin than the three Asian *D*. *melanogaster* stocks are, it is also far less resistant than strictly mycophagous *Drosophila* species [15], which puts *D*. *tripunctata* in an intermediate position on the way to strict mycophagy. Taken together, if pesticides really did cause α-amanitin resistance in *D*. *melanogaster*, the use of them may go far beyond their intended effects and may change ecosystems in the long term.

### Limitations

When we created the isochromosomal lines, we did not balance the X chromosome because we were working under the published assumption that α-amanitin resistance *D*. *melanogaster* is conferred by two dominantly acting alleles on the third chromosome [21,22]. It is therefore possible that alleles derived from the X chromosome of the multi-balancer stock exert epistatic effects on the second and third chromosomes of the isochromosomal lines, which could explain why Ama-KTT/M/5 is more resistant than Ama-KTT, even if the multi-balancer stock itself is highly susceptible to α-amanitin.

During the fecundity studies, we harvested more eggs than could be counted each day. Therefore, we stored the egg-lay vials at 4°C immediately after they were collected, which killed the eggs. It was thus not possible to assess egg fertility and offspring vigor in response to the toxin. Future studies should test if the higher amounts of eggs that result from higher α-amanitin concentrations also give rise to a larger number of viable offspring, or if the eggs show a higher mortality in response to increasing toxin concentrations.

Although courtship can lead to reduced longevity in males [59], we did not accompany excessive experimental males with white-eyed *w*[1118] females because doing so would have interfered with our fecundity studies.

### Future research

In this study, we learned that all three Asian stocks display the same qualitative, but different quantitative responses to α-amanitin. Thus, the present research lays the foundation for molecular studies that can reveal the underlying causes for the observed quantitative variations in α-amanitin resistance in the three *D*. *melanogaster* stocks. One way to link the quantitative resistance phenotypes to the resistance-conferring genes would be to perform a microarray study, which includes larvae of all six isochromosomal lines. We already know that four candidate mechanisms are responsible for the resistance phenotype of Ama-KTT/M/2 larvae [26]. Thus, our isochromosomal lines could be a valuable resource to verify the most important candidate genes, which can then be tested by the transgenic rescue approach and/or mutagenesis. A similar microarray could also be performed in adults to test if stress response pathways are activated by the possibly remnant α-amanitin, which may be the cause for the increased fecundity and decreased longevity. Thus, future studies should aim for a better understanding of the molecular mechanisms that cause α-amanitin resistance and how it could persist over decades in the absence of selective pressure.

It would also be very interesting to test what factors caused the cross-resistance to α-amanitin in the first place. *Cyp6a2* is one of the best candidate genes for conferring resistance in Ama-KTT/M/2 larvae [26]. The CYP6A2 enzyme has been shown to metabolize organophosphorous [60] and organochlorine [27,60] insecticides. Thus, dose-response studies using such substances could shed light on the chemicals that caused the cross-resistance to α-amanitin in the three Asian stocks in their natural habitats more than five decades ago.

## Conclusions

Our observations collectively suggest that α-amanitin resistance in the three Asian *D*. *melanogaster* stocks Ama-KTT, Ama-MI, and Ama-KLM has evolved as cross-resistance that has no apparent fitness costs. Our data further confirm the conclusion of our recent microarray study [26] that α-amanitin resistance is a quantitative trait, rather than conferred by two dominantly acting loci on chromosome 3. The α-amanitin resistance phenotype is both interesting and important because it is likely a cross-resistance to agricultural pesticides, which suggests that pesticides may have unintentional effects on non-pest species and thus on entire ecosystems. In contrast to super-resistant mycophagous *Drosophila* species, low α-amanitin concentrations negatively influence *D*. *melanogaster*'s larva-to-adult developmental time, pre-adult viability, adult body size, and adult longevity, while the toxin increases female fecundity. Although *D*. *melanogaster* is not a pest, the long-term persistence of the resistance phenotype and the positive effects of α-amanitin on female fecundity are somewhat alarming.

## Materials and Methods

### Fly stocks

All *Drosophila melanogaster* (Meigen) stocks were maintained at room temperature on standard food containing cornmeal, granulated sugar, Brewer’s yeast, agar, and methylparaben as antifungal agent. The wild type stocks Canton-S and Oregon-R, the *white* mutant *w*[1118], and the multi-balancer stock *w*[1118]/Dp(1;Y)*y*[+]; CyO/*nub*[1] *b*[1] *sna*[Sco] *lt*[1] *stw*[3]; MKRS/TM6B, *Tb*[1] were obtained from the Bloomington Stock Center, Bloomington, IN, USA (stocks #1, #5, #3605, and #3703, respectively). The stocks Ama-KTT (#14021-0231.07), Ama-MI (#14021-0231.06), and Ama-KLM (#14021-0231.04) were shown to be resistant to α-amanitin in 1982 [22] and obtained from the *Drosophila* Species Stock Center at the University of California, San Diego, CA, USA. Ama-KTT and Ama-MI were originally collected in 1968 in Kenting (Taiwan) and in Mysore (India), respectively. Ama-KLM is the oldest of the three α-amanitin-resistant stocks and was collected in 1962 in Kuala Lumpur (Malaysia).

### Generation of the isochromosomal lines

Because Ama-KTT, Ama-MI, and Ama-KLM were maintained the absence of selective pressure to toxins in the stock center over the past five decades, the stocks could have lost or become heterozygous for some of the α-amanitin resistance-causing alleles. To create flies homozygous for the resistance-conferring alleles that remained in these stocks, we created isochromosomal lines that are isogenic for the second and third chromosomes (Fig 2). In order to guarantee that we collect most or all alleles, we started with one highly α-amanitin-resistant female of each stock that survived the following concentrations: Ama-KTT: 5 μg α-amanitin per g of food, Ama-MI: 7 μg α-amanitin per g of food, and Ama-KLM: 4 μg α-amanitin per g of food. We chose two resulting isochromosomal lines from each original α-amanitin-resistant stock to further investigate the resistance-causing alleles. The Ama-MI/**T**/6 isochromosomal line differs from the other stocks by its third chromosome being balanced over the **T**M6B, *Tb* chromosome in the F1 generation, while the other five isochromosomal lines Ama-KTT/**M**/2, Ama-KTT/**M**/5, Ama-MI/**M**/2, Ama-KLM/**M**/5, and Ama-KLM/**M**/7 were balanced over **M**KRS. The isochromosomal lines were selected for three subsequent generations against the white eye color that was introduced by the X-chromosome of the multi-balancer stock, until all isochromosomal lines were purely red-eyed.

### Dose-response studies of the fly stocks to α-amanitin

In order to quantify and compare the levels of α-amanitin resistance of the *D*. *melanogaster* stocks, dose-response experiments were performed, which measured the survival from freshly-hatched first-instar larvae to adulthood. Flies able to completely hatch from their pupae were counted as survivors. The α-amanitin-resistant stocks Ama-KTT, Ama-MI, Ama-KLM, and their isochromosomal derivates were tested on a total of 11 α-amanitin concentrations, using 0 to 10 μg of α-amanitin per g of food in 1 μg increments. The α-amanitin-sensitive wild type stocks Canton-S and Oregon-R, and the multi-balancer stock *w*[1118]/Dp(1;Y)*y*[+]; CyO/*nub*[1] *b*[1] *sna*[Sco] *lt*[1] *stw*[3]; MKRS/TM6B, *Tb*[1] were initially tested on five concentrations ranging from 0 to 4 μg of α-amanitin per g of food in 1μg increments. However, because they survived only the 0-toxin concentration, these stocks were further tested on 0, 0.025, 0.05, 0.075, 0.1, 0.25, and 0.375 μg of α-amanitin per g of food. α-Amanitin was purchased from Sigma-Aldrich, St. Louis, MO, USA.

Flies of mixed sexes were allowed to lay eggs on molasses agar caps that contained a streak of fresh Baker’s yeast paste at 25°C, 70% humidity, and a 12:12 hour day/night cycle in a *Drosophila* chamber (Model GSDR-36VL) from Geneva Scientific, Fontana, WI, USA. The yeast was removed prior to larval hatching. Freshly hatched first-instar larvae were placed in groups of ten into 2 mL plastic test tubes (USA Scientific, Orlando, FL, USA), each containing 500 mg of non-toxic or poisoned food and two small air holes in the lid. The food consisted of 125 mg dry, instant *Drosophila* medium (Carolina Biological, Burlington, NC, USA) and 375 μL sterile Milli-Q water with or without dissolved α-amanitin. Ten tubes were prepared for each toxin concentration and experimental replicate, resulting in 100 larvae for each concentration and a total of 1,100 larvae per experiment. Three high-quality dose-response experiments, in which the 0-toxin concentration survival rate was at least 80%, were used to calculate the LC_50_ of each fly stock. The standard error of the mean (s.e.m.) was calculated for each concentration by sampling the data points of all 30 vials of every concentration. The LC_50_ was calculated using scatter plots and the logarithmic trend line function in Microsoft Excel.

### Thorax measurements, fecundity, and longevity measurements

Surviving flies of the dose-response experiments were collected daily within 24 hours of hatching. To measure thorax lengths as an indicator of developmental retardation caused by the different α-amanitin concentrations, the flies were anesthetized using CO_2_. Thorax lengths were measured from the tip of the scutellum to the base of the neck while the flies were lying on one side [61], using an Olympus SZX16 dissection microscope, an Olympus DP72 camera, and cellSens Standard 1.3 software (Olympus, Center Valley, PA, USA).

For the fecundity and longevity tests, the flies were kept in the absence of α-amanitin in 25 x 95 mm *Drosophila* plastic vials (VWR International, Radnor, PA, USA) filled with 5 mL of molasses agar and a streak of Baker’s yeast paste. The flies were housed in small groups consisting of an equal number of males and females that hatched on the same day from the same toxin concentration. Because females without male partners live longer than females in the presence of males [38,62], white-eyed *w*[1118] males were added to the experimental females who were lacking male partners to balance the male-to-female ratio across all experiments. Because of their different eye color, the *w*[1118] males could be easily excluded from the survival counts. Every day throughout their lifespan, all survivors were transferred to new molasses vials with fresh yeast paste. The eggs in the vacated vials were first stored at 4°C and then counted to assess the daily fecundity of the females in response to different α-amanitin concentrations. In order to test if α-amanitin eaten during their larval life shortens the lifespan of the adults, the amount of the dead flies and their sexes were recorded daily.

### Statistical analyses

Microsoft Excel was used to create the graphs and perform the one-way ANOVA analyses. A logarithmic trend line was used to calculate the LC_50_ values.

## References

[1] Pimentel D, Acquay H, Biltonen M, Rice P, Silva M, Nelson J et al (1992) Environmental and economic costs of pesticide use. Bioscience 42: 750–760.

[2] Liu H, Cupp EW, Micher KM, Guo A, Liu N (2004) Insecticide resistance and cross-resistance in Alabama and Florida strains of Culex quinquefasciatus. J Med Entomol 41: 408–413. 15185942 10.1603/0022-2585-41.3.408

[3] Brengues C, Hawkes NJ, Chandre F, McCarroll L, Duchon S, Guillet P et al (2003) Pyrethroid and DDT cross-resistance in Aedes aegypti is correlated with novel mutations in the voltage-gated sodium channel gene. Med Vet Entomol 17: 87–94. 12680930 10.1046/j.1365-2915.2003.00412.x

[4] Mota-Sanchez D, Hollingworth RM, Grafius EJ, Moyer DD (2006) Resistance and cross-resistance to neonicotinoid insecticides and spinosad in the Colorado potato beetle, Leptinotarsa decemlineata (Say) (Coleoptera: Chrysomelidae). Pest Manag Sci 62: 30–37. 16206238 10.1002/ps.1120

[5] Rauch N, Nauen R (2003) Identification of biochemical markers linked to neonicotinoid cross resistance in Bemisia tabaci (Hemiptera: Aleyrodidae). Arch Insect Biochem Physiol 54: 165–176. 14635178 10.1002/arch.10114

[6] Tabashnik BE, Finson N, Johnson MW, Heckel DG (1994) Cross-resistance to Bacillus thuringiensis toxin CryIF in the diamondback moth (Plutella xylostella). Appl Environ Microbiol 60: 4627–4629. 16349471 10.1128/aem.60.12.4627-4629.1994 PMC202035

[7] Wei Y, Appel AG, Moar WJ, Liu N (2001) Pyrethroid resistance and cross-resistance in the German cockroach, Blattella germanica (L). Pest Manag Sci 57: 1055–1059. 11721523 10.1002/ps.383

[8] Liu N, Yue X (2000) Insecticide resistance and cross-resistance in the house fly (Diptera: Muscidae). J Econ Entomol 93: 1269–1275. 10985042 10.1603/0022-0493-93.4.1269

[9] Daborn PJ, Yen JL, Bogwitz MR, Le Goff G, Feil E, Jeffers S et al (2002) A single P450 allele associated with insecticide resistance in Drosophila. Science 297: 2253–2256. 12351787 10.1126/science.1074170

[10] Daborn P, Boundy S, Yen J, Pittendrigh B, Ffrench-Constant R (2001) DDT resistance in Drosophila correlates with Cyp6g1 over-expression and confers cross-resistance to the neonicotinoid imidacloprid. Mol Genet Genomics 266: 556–563. 11810226 10.1007/s004380100531

[11] Bloomquist JR (1994) Cyclodiene resistance at the insect GABA receptor/chloride channel complex confers broad cross resistance to convulsants and experimental phenylpyrazole insecticides. Arch Insect Biochem Physiol 26: 69–79. 8054658 10.1002/arch.940260106

[12] Vetter J (1998) Toxins of Amanita phalloides. Toxicon 36: 13–24. 9604278 10.1016/s0041-0101(97)00074-3

[13] Benjamin DA (1995) Mushrooms: poisons and panaceas: A handbook for naturalists, mycologists, and physicians New York: W.H. Freeman and Company.

[14] Lindell TJ, Weinberg F, Morris PW, Roeder RG, Rutter WJ (1970) Specific inhibition of nuclear RNA polymerase II by alpha-amanitin. Science 170: 447–449. 4918258 10.1126/science.170.3956.447

[15] Jaenike J, Grimaldi DA, Sluder AE, Greenleaf AL (1983) Alpha-amanitin tolerance in mycophagous Drosophila. Science 221: 165–167. 17769215 10.1126/science.221.4606.165

[16] Stump AD, Jablonski SE, Bouton L, Wilder JA (2011) Distribution and mechanism of alpha-amanitin tolerance in mycophagous Drosophila (Diptera: Drosophilidae). Environ Entomol 40: 1604–1612. 10.1603/EN11136 22217779

[17] Jaenike J (1985) Parasite pressure and the evolution of amanitin tolerance in Drosophila. Evolution 39: 1295–1301. 28564265 10.1111/j.1558-5646.1985.tb05695.x

[18] Spicer GS, Jaenike J (1996) Phylogenetic analysis of breeding site use and alpha-amanitin tolerance within the Drosophila quinaria species group. Evolution 50: 2328–2337. 28565683 10.1111/j.1558-5646.1996.tb03620.x

[19] Perlman SJ, Jaenike J (2003) Infection success in novel hosts: an experimental and phylogenetic study of Drosophila-parasitic nematodes. Evolution 57: 544–557. 12703944 10.1111/j.0014-3820.2003.tb01546.x

[20] Perlman SJ, Spicer GS, Shoemaker DD, Jaenike J (2003) Associations between mycophagous Drosophila and their Howardula nematode parasites: a worldwide phylogenetic shuffle. Mol Ecol 12: 237–249. 12492892 10.1046/j.1365-294x.2003.01721.x

[21] Begun DJ, Whitley P (2000) Genetics of alpha-amanitin resistance in a natural population of Drosophila melanogaster. Heredity 85: 184–190. 11012721 10.1046/j.1365-2540.2000.00729.x

[22] Phillips JP, Willms J, Pitt A (1982) Alpha-amanitin resistance in three wild strains of Drosophila melanogaster. Canad J Genet Cytol 24: 151–162. 6812932 10.1139/g82-014

[23] Hallen HE, Adams GC, Eicker A (2002) Amatoxins and phallotoxins in indigenous and introduced South African Amanita species. S Afr J Bot 68: 322–326.

[24] Hallen HE, Luo H, Scott-Craig JS, Walton JD (2007) Gene family encoding the major toxins of lethal Amanita mushrooms. Proc Natl Acad Sci U S A 104: 19097–19101. 18025465 10.1073/pnas.0707340104 PMC2141914

[25] Walton JD, Hallen-Adams HE, Luo H (2010) Ribosomal biosynthesis of the cyclic peptide toxins of Amanita mushrooms. Biopolymers 94: 659–664. 10.1002/bip.21416 20564017 PMC4001729

[26] Mitchell CL, Saul MC, Lei L, Wei H, Werner T (2014) The mechanisms underlying alpha-amanitin resistance in Drosophila melanogaster: A microarray analysis. PLoS One 9: e93489 10.1371/journal.pone.0093489 24695618 PMC3973583

[27] Amichot M, Tares S, Brun-Barale A, Arthaud L, Bride JM, Berge JB (2004) Point mutations associated with insecticide resistance in the Drosophila cytochrome P450 Cyp6a2 enable DDT metabolism. Eur J Biochem 271: 1250–1257. 15030474 10.1111/j.1432-1033.2004.04025.x

[28] Brun A, Cuany A, Le Mouel T, Berge J, Amichot M (1996) Inducibility of the Drosophila melanogaster cytochrome P450 gene, CYP6A2, by phenobarbital in insecticide susceptible or resistant strains. Insect Biochem Mol Biol 26: 697–703. 8995791 10.1016/s0965-1748(96)00036-7

[29] Daborn PJ, Lumb C, Boey A, Wong W, Ffrench-Constant RH, Batterham P (2007) Evaluating the insecticide resistance potential of eight Drosophila melanogaster cytochrome P450 genes by transgenic over-expression. Insect Biochem Mol Biol 37: 512–519. 17456446 10.1016/j.ibmb.2007.02.008

[30] Festucci-Buselli RA, Carvalho-Dias AS, de Oliveira-Andrade M, Caixeta-Nunes C, Li HM, Stuart JJ et al (2005) Expression of Cyp6g1 and Cyp12d1 in DDT resistant and susceptible strains of Drosophila melanogaster. Insect Mol Biol 14: 69–77. 15663776 10.1111/j.1365-2583.2005.00532.x

[31] Kalajdzic P, Oehler S, Reczko M, Pavlidi N, Vontas J, Hatzigeorgiou AG et al (2012) Use of mutagenesis, genetic mapping and next generation transcriptomics to investigate insecticide resistance mechanisms. PLoS One 7: e40296 10.1371/journal.pone.0040296 22768270 PMC3386967

[32] Le Goff G, Hilliou F, Siegfried BD, Boundy S, Wajnberg E, Sofer L et al (2006) Xenobiotic response in Drosophila melanogaster: sex dependence of P450 and GST gene induction. Insect Biochem Mol Biol 36: 674–682. 16876710 10.1016/j.ibmb.2006.05.009

[33] Grimaldi D, Jaenike J (1984) Competition in natural populations of mycophagous Drosophila. Ecology 65: 1113–1120.

[34] Partridge L, Barrie B, Fowler K, French V (1994) Evolution and development of body size and cell size in Drosophila melanogaster in response to temperature. Evolution 48: 1269–1276. 28564446 10.1111/j.1558-5646.1994.tb05311.x

[35] Bubliy OA, Loeschcke V (2005) Correlated responses to selection for stress resistance and longevity in a laboratory population of Drosophila melanogaster. J Evol Biol 18: 789–803. 16033550 10.1111/j.1420-9101.2005.00928.x

[36] Fellowes MDE, Kraaijeveld AR, Godfray HCJ (1999) The relative fitness of Drosophila melanogaster (Diptera, Drosophilidae) that have successfully defended themselves against the parasitoid Asobara tabida (Hymenoptera, Braconidae). J Evolution Biol 12: 123–128.

[37] Norry FM, Loeschcke V (2002) Temperature-induced shifts in associations of longevity with body size in Drosophila melanogaster. Evolution 56: 299–306. 11926498 10.1111/j.0014-3820.2002.tb01340.x

[38] Fowler K, Partridge L (1989) A cost of mating in female fruit flies. Nature 338: 760–761.

[39] Leips J, Mackay TF (2000) Quantitative trait loci for life span in Drosophila melanogaster: interactions with genetic background and larval density. Genetics 155: 1773–1788. 10924473 10.1093/genetics/155.4.1773 PMC1461186

[40] Spencer CC, Howell CE, Wright AR, Promislow DE (2003) Testing an 'aging gene' in long-lived drosophila strains: increased longevity depends on sex and genetic background. Aging Cell 2: 123–130. 12882325 10.1046/j.1474-9728.2003.00044.x PMC3991309

[41] McCart C, Buckling A, Ffrench-Constant RH (2005) DDT resistance in flies carries no cost. Curr Biol 15: R587–R589. 16085476 10.1016/j.cub.2005.07.054

[42] McCart C, Ffrench-Constant RH (2008) Dissecting the insecticide-resistance-associated cytochrome P450 gene Cyp6g1. Pest Manag Sci 64: 639–645. 10.1002/ps.1567 18338338

[43] Tuno N, Takahashi KH, Yamashita H, Osawa N, Tanaka C (2007) Tolerance of Drosophila flies to ibotenic acid poisons in mushrooms. J Chem Ecol 33: 311–317. 17195114 10.1007/s10886-006-9228-3

[44] Peters TM, Barbosa P (1977) Influence of population density on size, fecundity, and developmental rate of insects in culture. Annu Rev Entomol 22: 431–450.

[45] Norry FM, Loeschcke VR (2002) Longevity and resistance to cold stress in cold-stress selected lines and their controls in Drosophila melanogaster. J Evolution Biol 15: 775–783.

[46] Vijendravarma RK, Kraaijeveld AR, Godfray HC (2009) Experimental evolution shows Drosophila melanogaster resistance to a microsporidian pathogen has fitness costs. Evolution 63: 104–114. 10.1111/j.1558-5646.2008.00516.x 18786186

[47] Smith JM (1958) The effects of temperature and egg-laying on the longevity of Drosophila subobscura. J Exp Biol 35: 832–842.

[48] Javois J, Tammaru T (2004) Reproductive decisions are sensitive to cues of life expectancy: the case of a moth. Anim Behav 68: 249–255.

[49] Adamo SA (1999) Evidence for adaptive changes in egg laying in crickets exposed to bacteria and parasites. Anim Behav 57: 117–124. 10053078 10.1006/anbe.1998.0999

[50] Liu ZW, Han ZJ (2006) Fitness costs of laboratory-selected imidacloprid resistance in the brown planthopper, Nilaparvata lugens Stal. Pest Manag Sci 62: 279–282. 16475223 10.1002/ps.1169

[51] Hurd H, Taylor PJ, Adams D, Underhill A, Eggleston P (2005) Evaluating the costs of mosquito resistance to malaria parasites. Evolution 59: 2560–2572. 16526504 PMC1602058

[52] Webster JP, Woolhouse MEJ (1999) Cost of resistance: relationship between reduced fertility and increased resistance in a snail-schistosome host-parasite system. P Roy Soc B-Biol Sci 266: 391–396.

[53] Eritja R, Chevillon C (1999) Interruption of chemical mosquito control and evolution of insecticide resistance genes in Culex pipiens (Diptera: Culicidae). J Med Entomol 36: 41–49. 10071491 10.1093/jmedent/36.1.41

[54] Chevillon C, Bourguet D, Rousset F, Pasteur N, Raymond M (1997) Pleiotropy of adaptive changes in populations: comparisons among insecticide resistance genes in Culex pipiens. Genet Res 70: 195–203. 9494436 10.1017/s0016672397003029

[55] Raymond B, Sayyed AH, Wright DJ (2005) Genes and environment interact to determine the fitness costs of resistance to Bacillus thuringiensis. P Roy Soc B-Biol Sci 272: 1519–1524. 16011928 10.1098/rspb.2005.3103 PMC1559826

[56] Gassmann AJ, Stock SP, Carriere Y, Tabashnik BE (2006) Effect of entomopathogenic nematodes on the fitness cost of resistance to Bt toxin crylac in pink bollworm (Lepidoptera: Gelechiidae). J Econ Entomol 99: 920–926. 16813331 10.1603/0022-0493-99.3.920

[57] Jaenike J (1989) Genetic population structure of Drosophila tripunctata—Patterns of variation and covariation of traits affecting resource use. Evolution 43: 1467–1482. 28564234 10.1111/j.1558-5646.1989.tb02597.x

[58] Jaenike J, Grimaldi D (1983) Genetic variation for host preference within and among populations of Drosophila tripunctata. Evolution 37: 1023–1033. 28563549 10.1111/j.1558-5646.1983.tb05630.x

[59] Cordts R, Partridge L (1996) Courtship reduces longevity of male Drosophila melanogaster. Anim Behav 52: 269–278.

[60] Dunkov BC, Guzov VM, Mocelin G, Shotkoski F, Brun A, Amichot M et al (1997) The Drosophila cytochrome P450 gene Cyp6a2: structure, localization, heterologous expression, and induction by phenobarbital. DNA Cell Biol 16: 1345–1356. 9407006 10.1089/dna.1997.16.1345

[61] Karan D, Morin JP, Moreteau B, David JR (1998) Body size and developmental temperature in Drosophila melanogaster: Analysis of body weight reaction norm. J Therm Biol 23: 301–309.

[62] Chapman T, Liddle LF, Kalb JM, Wolfner MF, Partridge L (1995) Cost of mating in Drosophila melanogaster females is mediated by male accessory gland products. Nature 373: 241–244. 7816137 10.1038/373241a0

